# Authors’ reply

**Published:** 2010

**Authors:** Rishi Nayyar, Prabhjot Singh, Narmada P. Gupta, Ashok K. Hemal, Prem N. Dogra, Amlesh Seth, Rajeev Kumar

**Affiliations:** Department of Urology, All India Institute of Medical Sciences, New Delhi, India

Dear Editor,

We thank Dr. Gautam[1] for his comments on the subject.[2] We totally agree with his observation and sincerely feel that it’s high time to develop uropathology as a specialized branch. urological pathology especially prostate has caught every ones eye all over the world and accurate pre-treatment pathological staging can only help in optimal treatment modality selection be it radical surgery, radiation, minimally invasive treatments or surveillance. Apart from this we do not feel any clarifications or elaborations are needed on this subject.
